# *Aprostocetus nitens* (Hymenoptera: Eulophidae), an Ectoparasitoid Proposed for Biological Control of the Destructive Erythrina Gall Wasp, *Quadrastichus erythrinae*, in Hawaiʻi

**DOI:** 10.3390/insects16050519

**Published:** 2025-05-14

**Authors:** Mohsen M. Ramadan, Juliana A. Yalemar, Daniel Rubinoff, Mark G. Wright, Aimé H. Bokonon-Ganta, Xingeng Wang

**Affiliations:** 1 Hawaii Department of Agriculture, Division of Plant Industry, 1428 South King Street, Honolulu, HI 96814, USA; 2 Department of Plant and Environmental Protection Sciences, University of Hawaiʻi at Mānoa, 3050 Maile Way, Honolulu, HI 96822, USA; rubinoff@hawaii.edu (D.R.); markwrig@hawaii.edu (M.G.W.); 3 Faculty of Agronomic Sciences, University of Abomey-Calavi, Jéricho, Cotonou 03 B.P. 2819, Benin; aimehbg2@gmail.com; 4 USDA—ARS Beneficial Insects Introduction Research Unit, 501 South Chapel Street, Newark, DE 19713, USA; xingeng.wang@usda.gov

**Keywords:** biological control, tri-trophic association, *Erythrina sandwicensis*, Eulophidae, Eurytomidae, Hawaii, wiliwili

## Abstract

*Aprostocetus nitens* Prinsloo & Kelly (Hymenoptera: Eulophidae) was identified as one of four hymenopteran ectoparasitoids utilizing three erythrina gall wasps, *Quadrastichus bardus*, *Q. erythrinae*, and *Q. gallicola*) (Hymenoptera: Eulophidae) in the native eastern Africa. In Hawaiʻi, the eurytomid wasp, *Eurytoma erythrinae* Gates & Delvare (Hymenoptera: Eurytomidae), was introduced and approved for statewide release in 2008 to control the erythrina gall wasp (EGW) *Q. erythrinae* Kim. EGW has devastated the wiliwili trees, *Erythrina sandwicensis* Degener (Fabaceae), an ecologically and culturally important native Hawaiian tree species. However, the parasitoid’s impact on the galled inflorescences and shoots was not adequate to ensure adequate seed set and maturation for successful tree recruitment. *Aprostocetus nitens* was thus evaluated as a prospective natural enemy to enhance the biological control of EGW to further protect the wiliwili trees in Hawaiʻi. Both choice and no-choice host specificity tests were conducted on seven non-target gall formers in the Hawaii Department of Agriculture, Insect Containment Facility, and showed that the parasitoid was extremely specific to EGW. The potential for competition between this parasitoid and the established *E. erythrinae* was also investigated, showing that the release of a second parasitoid will potentially complement the success of the eurytomid wasp for control of EGW. Unlike what was found in the native region, the Hawaiian laboratory colony is thelytokous, producing only female offspring. The life cycle took 20.1 ± 0.28 days under the laboratory conditions. Non-ovipositing female survived for 102.5 ± 2.9 days when fed honey and laid eggs for 25.1 ± 2.3 days with average fecundity of 156.7 ± 22.3 offspring/female. This value is 3.9-fold higher than offspring produced by *E. erythrinae*. *Aprostocetus nitens*, host specificity, competition with *E. erythrinae*, and its tri-trophic association with 15 *Erythrina* host plants and 5 gall wasp assemblages in the native African regions were defined. Implications to reduce frequent galls on the native *Erythrina* plants and likely domination over *E. erythrinae*, are discussed.

## Simple Summary

*Aprostocetus nitens*, an eulophid parasitic wasp native to eastern Africa, is being evaluated in Hawaiʻi to control the destructive erythrina gall wasp (EGW), *Quadrastichus erythrinae*, which has devastated the native wiliwili trees, *Erythrina sandwicensis.* Host range assessment of six non-target gall-forming insects using choice and no-choice tests revealed that *A. nitens*, highly specific to EGW, targets its immature stages on leaves, flowers, and pods. It is expected to complement the existing biological control agent, the eurytomid wasp, *Eurytoma erythrinae*, to further reduce EGW populations and protect wiliwili trees on the Hawaiian Islands. We also report on the diverse tri-trophic associations of *Erythrina* host plants, gall wasps, and their parasitoid guilds in the native African regions.

## 1. Introduction

**Figure 1:**
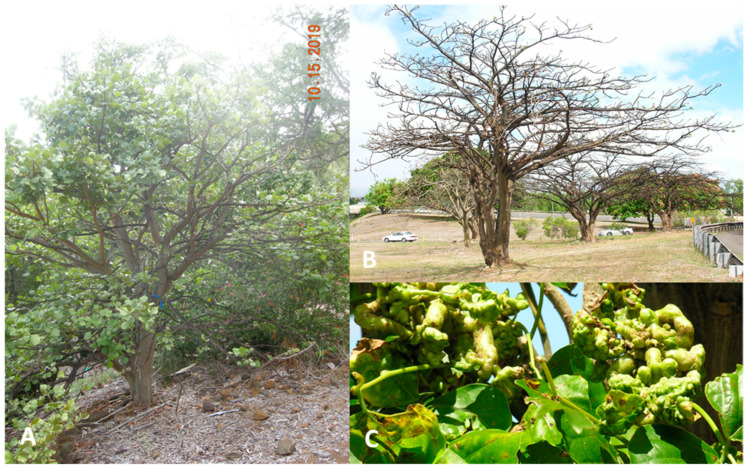
Recovery of the *Erythrina sandwicensis* trees after the invasion of the *Erythrina* gall-forming wasp (EGW), *Quadrastichus erythrinae*, 2019 (**A**); *Erythrina variegata* trees severely damaged in 2005 (**B**); infestation of *E. variegata* by EGW resulted in galled leaves and stems before the biocontrol introduction of the parasitoid *Eurytoma erythrinae* (**C**).

In April 2005, the Erythrina gall-forming wasp (EGW), *Quadrastichus erythrinae* Kim (Hymenoptera: Eulophidae), was detected on *Erythrina* trees (Fabaceae) on the island of Oahu (21.4389° N, 158.0001° W). Africa was confirmed as the area of origin of *Q. erythrinae,* and Tanzania is the putative source of the populations that invaded Hawaiʻi. Once the wasp was established, it was dispersed via wind and through human activities such as hiking and shipping. EGW rapidly spread and devastated *Erythrina* trees throughout the Hawaiian Islands. Thousands of *Erythrina variegata* L. trees used primarily for landscaping and windbreaks were all decimated throughout the state (Figure 1). The native plant, *Erythrina sandwicensis* O.Deg., native Hawaiian name “wiliwili”, is a highly valued dominant species of Hawaii’s lowland dry forests and was severely damaged with more than 40% tree mortality recorded in some areas [1,2,3,4,5,6,7,8].

EGW is a minute wasp, ranging from 1–1.5 mm in length. The life cycle of EGW in Hawaiʻi is nearly 20 days, which includes the egg stage (4 d), the larval stage (10 d), and the pupal stage (6 d) (HDOA Plant Pest and Control Branch 2008 records). The female is synovigenic and emerges with about 60 mature eggs in its ovaries. A female wasp can lay up to 350 eggs by inserting them into the young tissues of the plant. The larvae and pupae develop within the tissues, resulting in the swelling and formation of galls on young leaves and petioles (Figure 1C). Each gall harbors one larva in one chamber [4]. Heavily galled leaves and stems result in loss of growth and vigor as photosynthesis is reduced and plant health declines. Severe infestations eventually lead to tree mortality (Figure 1B) [3].

Because EGW feeds within plant tissues, it was thought that systemic pesticides could be used to control infestations of this species. However, widespread use of this method is unreasonable, and frequent, long-term use of insecticides in natural areas is neither feasible nor advisable. Attempts in Honolulu to control EGW on iconic landscaping trees (*Erythrina variegata*) using systemic pesticides were unsuccessful, and eventually resulted in the plants being cut down, after thousands of dollars were spent on chemicals. Insecticidal control was discontinued. The wasp spread quickly to several species of *Erythrina* in the state, including the Brazilian coral tree, *Erythrina crista-galli* L., Indian coral tree, *E. variegata*, and wiliwili, *E. sandwicensis*. Wiliwili, a native Hawaiian species, showed greater than 40% mortality in some affected populations [9,10]. Severe infestations have also devastated native and introduced trees of the genus *Erythrina* in the Western Pacific and Hawaii (China, Guam, Hawaii, Japan, Malaysia, Philippines, Samoa, Singapore, Taiwan, and Thailand). Sanitation through pruning and chemical control measures has failed to contain this pest, and biological control is thought to be the only long-term solution [3,5,11,12,13,14,15].

**Figure 2:**
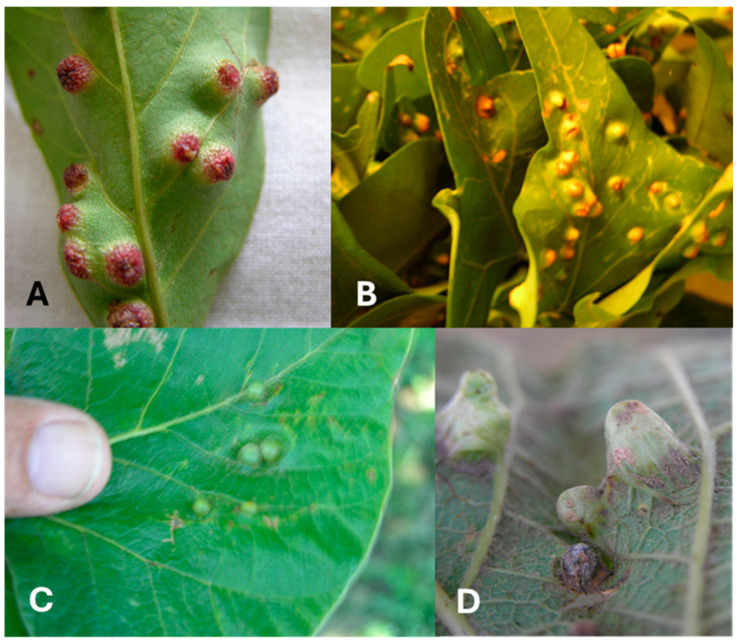
Galled *Erythrina* leaves in Africa: (**A**) *Erythrina crista-galli*; (**B**) *E. lysistemon* (18 galls per leaflet), SA; (**C**) gall shape, upper surface of *E. latissima* leaf (gall diameter 0.4 cm), SA; (**D**) gall shape (6 galls per leaf in two masses), lower surface of *E. latissima* leaf (midrib = 15 cm), SA.

Failed attempts to control EGW with conventional methods prompted the Hawaiʻi Department of Agriculture (HDOA), along with the University of Hawaii and other government agencies, to initiate a biological control project to examine species that would be appropriate biocontrol against EGW. Shortly thereafter, the survey team of five exploratory entomologists collected infested *Erythrina* leaves and stem samples from Tanzania, east Africa, and west Africa and shipped them to the HDOA Insect Quarantine Facility for parasitoid emergence [16]. *Erythrina* trees in Tanzania were very healthy and had low levels of gall insects (Figure 2). Gall abundance, sizes, and shapes vary on different *Erythrina* trees (Figure 2). Four main parasitoids known to attack EGW were collected during the exploratory surveys in East Africa in 2005–2007; they parasitized most of the wasp immature stages in the galls. These were ectoparasitoids including three eulophid parasitoids (*Aprostocetus exertus* LaSalle, *A. nitens* Prinsloo and Kelly, and *A. tritus* Prinsloo and Kelly) and the eurytomid parasitoid, *Eurytoma erythrinae* Gates and Delvare. *Eurytoma erythrinae* and *A. nitens* were amenable to laboratory rearing and showed potential as biocontrol agents. *Aprostocetus exertus* was not suitable for captive propagation owing to its male-biased sex ratio after four generations of rearing. *Aprostocetus tritus* was not reared [16,17,18,19].

After extensive evaluations at the HDOA Insect Containment Facility for host-specificity to confirm that *E. erythrinae* prefers to feed exclusively on EGW, field releases took place in November 2008. About 4000 individuals were released at various sites on the islands of Hawaiʻi, Kauaʻi, Maui, Molokaʻi, and Oʻahu. Within the span of a few months, the wiliwili trees began to show signs of recovery, with healthy new leaves and vigorous overall growth. By the second year after the release of *E. erythrinae*, more than 60% of young shoots were predominantly free of damage by EGW, tree canopies recovered each year after the release. By 2011, 90% of the sample trees had full canopy coverage (Figure 1A) [20,21,22,23].

**Figure 3:**
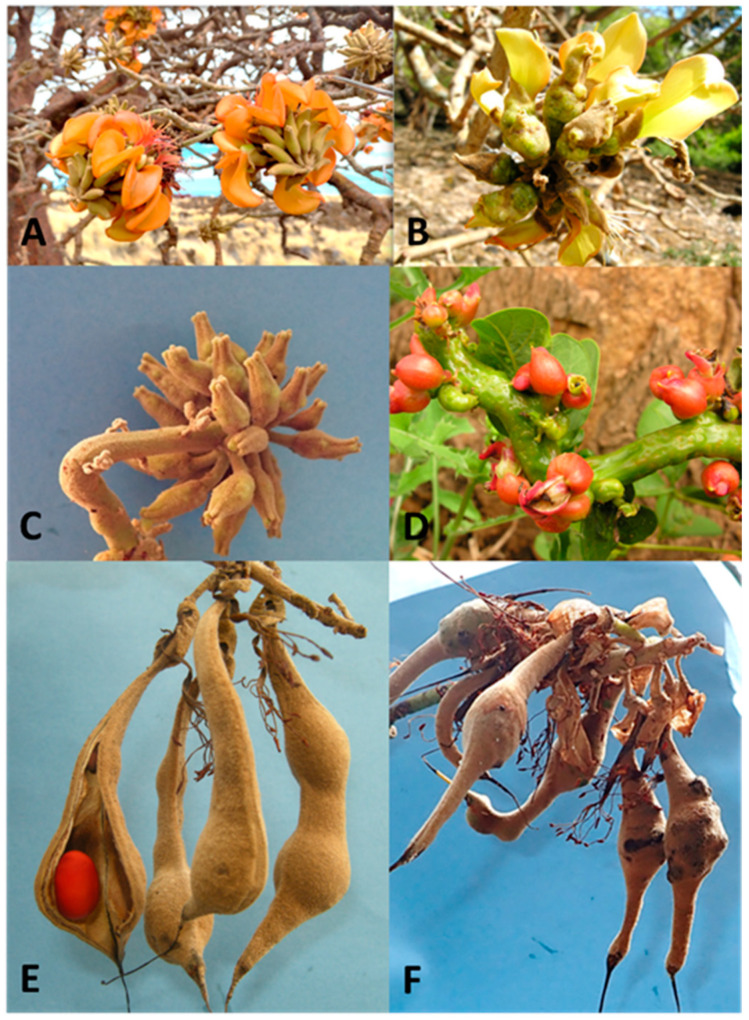
Infestation of *Erythrina* gall wasp on inflorescences and seed pods: (**A**) normal uninfested wiliwili red flowers before EGW; (**B**) infested yellow wiliwili flowers; (**C**) infested flower buds; (**D**) heavily infested flower buds of *Erythrina crista-galli*; (**E**) uninfested seed pods showing scarlet seed of wiliwili; (**F**) underdeveloped infested seed pods of wiliwili.

The progressive increase of flower and seed production indicated that the overall tree health of *E. sandwicensis* also improved [21]. Depending on the location, recent weather, and time of year, parasitism rates by *E. erythrinae* of EGW larvae inside galls range from 20–100%. Flowering and fruiting were restored, and the number of flowers increased each year post-release. However, 54% of the sampled inflorescences failed to form mature seeds due to gall wasp damage. Since the flowering season coincides with warm summer months (May–October), which is favorable for EGW development, the damage to wiliwili flowers continues. Most wiliwili seedlings are still unable to survive the damage of the pest. This adverse impact on flowers and seed pods production is not only an ecological concern, but also a cultural issue because Native Hawaiians value the scarlet wiliwili seeds for lei making (Figure 3E). Low production of viable seeds and seedling mortality remain threats to the future survival of *E. sandwicensis.* A potentially viable option is to seek another biocontrol agent that can augment the current impacts of *E. eythrinae*, and reduce the damage to reproductive structures and seedlings (Figure 3) [21,22].

**Figure 4:**
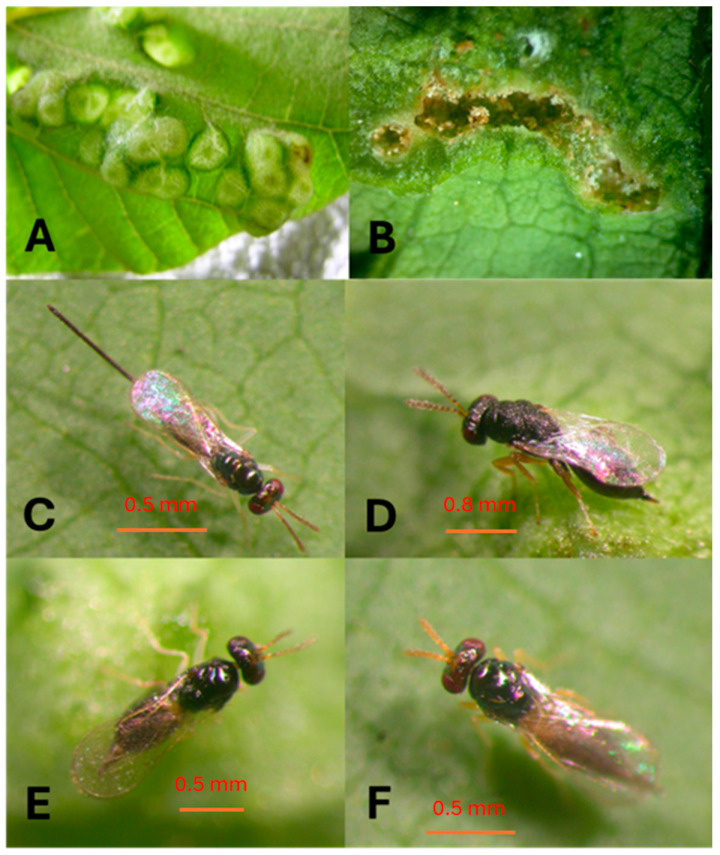
Major parasitoids of gall wasps in Africa: (**A**) galls in *Erythrina abyssinia*; (**B**) tunnel between galls made by *Eurytoma erythrinae* larva (larva removed); (**C**) habitus of *Aprostocetus exertus*; (**D**) habitus of *Eurytoma erythrinae*; (**E**) habitus of *Aprostocetus nitens*, dark strain ex. Kenya and Tanzania; (**F**) *Aprostocetus nitens* light colored abdomen, ex. Tanzania and South Africa.

Thus, despite the success of the release of *E. erythrinae*, it has been proposed that there was a need for a second species, *A. nitens*, to be released to enhance biocontrol and suppress EGW populations. Because *E. erythrinae* acts more as a predator than a parasitoid, with its larvae feeding on EGW galls and tunneling to feed on other larvae in adjacent galls, larger galls with many individual EGW are preferred for oviposition (Figure 4A,B). This feeding behavior excludes galled young *Erythrina* seedlings, as well as flowers and seed pods, where smaller galls tend to form at lower density, and effectively escape parasitism by *E. erythrinae* [22]. As a complementary biocontrol agent to *E. erythrinae*, HDOA thus proposed to release *A. nitens* from containment into the natural environment of the state of Hawaiʻi. Unlike *E. erythrinae*, *A. nitens* utilizes only one host individual to complete its development, and therefore, it can live on much smaller galls, such as those found in flowers, pods, and young seedlings (Figure 3). It is anticipated that the release of *A. nitens* will increase the likelihood of tree recruitment, and improve the overall survival of wiliwili.

Here, we report on foreign exploration for EGW parasitoids in Africa, the biology and host specificity testing of *A. nitens,* as well as the potential interaction between *A. nitens* and *E. erythrinae.* The tri-trophic interactions of the diverse *Erythrina* hosts, gall formers, and their associated parasitoids in Africa are also described.

## 2. Materials and Methods

### 2.1. Explorations and Origin of the Parasitoid Colony

Eight African countries were surveyed during this project by the HDOA and University of Hawaiʻi survey teams. East Africa (Kenya, Madagascar, Mozambique, South Africa, Tanzania) were surveyed in December 2005, January–June 2006, January–April 2007, and West Africa (Benin, Ghana, and Togo) were surveyed in May–June 2006 (see detailed collection information on Table S1). Samples of galled *Erythrina* leaves were shipped to the HDOA Insect Containment Facility (ICF). There, leaves were placed as layers on wire screens and paper towel in screened cages (30 × 30 × 60 cm, 70 mesh) for wasp emergence at (mean ± SEM, 21.8 ± 0.12 °C, 70.2 ± 2.4% RH, 12:12, D: L photoperiod). Specimens of emerging wasps were preserved in 70% alcohol for later identification. We used the Prinsloo and Kelly (2009) manuscript to identify the gall formers and parasitoids [19]. Voucher specimens were sent to La Salle (hymenopteran taxonomist, CSIRO, Pullenvale, QLD, Australia) and Prinsloo (Pretoria, South Africa) for confirmation of species identity. The characters of color and submarginal setae separated *Quadrastichs* from *Aprostocetus* parasitoids according to the key ([19], Figures S1 and S2). Diversity and distribution of the Hymenopterous gall inducers of native *Erythrina* were determined with the recovered parasitoids.

Voucher specimens of *Aprostocetus nitens* were deposited in the insect reference collection of the HDOA, the Bernice P. Bishop Museum, and UH insect collection, Honolulu, Oʻahu.

### 2.2. Propagation of Host Plants and EGW

*Erythrina variegata* was used as the host plant for EGW rearing in our tests due to its availability and rapid growth. Seeds were scarified using a grinding machine. Two seeds were planted in 10 cm square pots and kept in outdoor screened cages (76 × 88 × 127 cm, 70 mesh) to avoid contamination with any pest scales, mites, aphids, or thrips. Seedlings were watered every other day. In 6–8 weeks, seedlings were ready to be used in propagating gall insects; the ideal plants are ±45 cm tall, 2–3 branches (about 2 cm long), with new leaves. We set up each rearing cage for EGW with two uninfested *E. variegata* plants in saucers. Fifteen pairs of EGW were placed in the cage for 7 days until galls had formed on young leaves and petioles. Plants were then transferred to cages (42 × 42 × 62 cm, 70 mesh) and held in HDOA-ICF in the conditions described above and were watered every other day. Galled *E. variegata* seedlings were ready to be used for parasitoid propagation 14 days after exposure to EGW.

### 2.3. Rearing of A. nitens

Twenty adults of *A. nitens* were placed in each cage, containing four plants that were previously exposed to EGW for 14 days as described above. Only female adults were needed for inoculation since this parasitoid is a thelytokous strain producing only females under HDOA-ICF laboratory conditions. Female *A. nitens* lays eggs up to 30–60 days. Adults usually emerged within 20 days after exposure. If no emergence occurred after 28 days, the plants were inoculated again with new female *A. nitens*. Newly infested *Erythrina* plants with EGW galls were added every 2–3 weeks to ensure plants in the cage had a continuous supply of new galls available.

### 2.4. Longevity and Life-Time Fecundity of A. nitens

Longevity of non-ovipositing females was determined by collecting newly emerged adults of *A. nitens* and placing 5–10 females in clear plastic vials (27 mm Ø, 55 mm height). The vials were covered with a piece of muslin cloth material and secured with a ventilated plastic cap. Honey (SUE BEE^®^ SPUN^®^ (https://siouxhoney.com/sue-bee-spun-honey), Sioux City, IA, USA, accessed on 1 January 2025) was dotted on muslin cloth material for feeding the females. Females without access to honey were also measured for their longevity. The number of dying wasps was recorded daily until all perished. Tests were replicated ten times. The mean survivorship of 100 and 58 females was determined for honey-fed and starved treatments, respectively.

Fecundity tests were conducted to determine the number of progeny a female could produce in her lifetime. One *E. variegata* plant with 14-d-old galls was placed in a (30 × 30 × 60 cm, 70 mesh) aluminum cage with a newly emerged female *A*. *nitens*. The plant was exposed to a female parasitoid for 6–7 days, then the plant was removed and replaced with a new plant with new EGW galls until the female died. Exposed plants were held in a cage, and the emerged parasitoid offspring were counted and recorded daily. After all parasitoids emerged, galls were dissected to count the non-emerging wasps. This test was repeated seven times.

### 2.5. Host Specificity Testing of A. nitens

All host specificity tests for *A. nitens* were conducted in the HDOA-ICF. Both choice and no-choice tests were conducted to determine whether *A. nitens* would feed on non-target hosts in the presence or absence of its target host. Seven non-target gall-forming insects were tested (same species as used in trials of *E. erythrinae*, Nagamine et al. [23]): Hamakua pamakani gall fly, *Procecidochares alani* Steyskal, a biocontrol agent of *Ageratina riparia*; Maui pamakani gall fly, *Procecidochares utilis* Stone, a biocontrol agent of *Ageratina adenophora*; lantana gall fly, *Eutreta xanthochaeta* Aldrich, a biocontrol agent of *Lantana camara* (all Diptera: Tephritidae); banyan gall wasp, *Josephiella microcarpae* Beardsley & Rasplus (Hymenoptera: Agaonidae), pest of *Ficus microcarpa*, Chinese banyan; a native psyllid, (*Pariaconus* sp., Hemiptera: Psyllidae), on ʻōhiʻa lehua *Metrosideros polymorpha*; a eulophid wasp, *Ophelimus* sp. (Hymenoptera: Eulophidae), a pest of *Eucalyptus* sp.; and a scale insect, *Tectococcus ovatus* Hempel (Hemiptera: Eriococcidae), a biocontrol agent of strawberry guava, *Psidium cattleianum*. Whole plants or cuttings were used in the experiments for choice or no-choice tests.

In the choice tests, a plant or cutting harboring one of the seven non-target insects and an EGW-infested *Erythrina* plant were placed side-by-side in a screened aluminum cage (46 × 46 × 76 cm, 70 mesh). Ten newly emerged *A. nitens* females from the laboratory colony were placed in the cage with honey provided as a source of food for the adult parasitoids. Behavioral observations were conducted to record the number of visits by the 10 *A. nitens* to each plant or cutting and whether they were resting or attempting to oviposit. Six daily counts were made at successive hourly intervals for two consecutive days (8:00 a.m.–14:00 p.m.). Parasitoids were then removed after two weeks, and the test and control plants were placed in separate cages to await parasitoid emergence. After one month, galls from each test plant were dissected and examined under a microscope to determine whether parasitism had taken place. Procedures were similar for the no-choice test, except that the parasitoids were given only one plant or cutting infested with one of the seven non-target gall-formers. As a control, *Erythrina* plants infested with EGW were exposed to the parasitoids in separate cages. There were three replicates of each insect tested in both choice and no-choice tests.

### 2.6. Intersepcific Competition Between A. nitens and E. erythrinae

Competition trials were performed in the HDOA-ICF. Because *A. nitens* is proposed to be released to complement the beneficial impacts made by the introduction of *E. erythrinae*, multiple parasitism trials were performed to determine (1) the level of *Erythrina* gall wasp parasitism by the two parasitoids when either is used alone or when both are used concurrently; (2) if the sequence of *Erythrina* gall wasp exposure to the two parasitoids will have differential effects on their progeny; and (3) if interspecific competition would reduce the efficiency by each or both parasitoids. The experimental treatments were as follows:

1. Control: Plants with *Erythrina* gall wasp only, no parasitoids;
2. Galled *Erythrina* were exposed to 10 females of *E. erythrinae* only, for 72 h;
3. Galled *Erythrina* were exposed to 10 *A. nitens* only, for 72 h;
4. Galled *Erythrina* were exposed to 10 females of *E. erythrinae* and 10 females of *A. nitens* concurrently for 72 h;
5. Galled *Erythrina* were exposed to 10 females of *E. erythrinae* for 72 h. Then, after 4 days, the same plants were exposed to 10 *A. nitens* for 72 h;
6. Galled *Erythrina* were exposed to 10 females of *A. nitens* for 72 h. Then, after 4 days, the same plants were exposed to 10 females of *E. erythrinae* for 72 h.

For each of the treatments 2–6, gravid females of *E. erythrinae* (7 d-old) and/or *A. nitens* (3 d-old) were introduced to galled *E. variegata.* Before the introduction of the two parasitoids, the level of galling was rated to ensure that both were exposed to similar levels of galling. After this exposure time, the plants were removed from the cages and held for 4 weeks to rear out the subsequently emerging parasitoids or EGW. All emerging adults were captured and counted. Fourteen days after exposure to the parasitoids, a subsample of the galls was removed from each plant and dissected to determine the number of parasitoids developing within the galls or to determine the parasitism rate of each parasitoid. The experiment was repeated five times.

Treatments 2–4 comprise an additive-series design where the number of each parasitoid species was the same in the single- and two-species treatments at the same host density, so that possible intraspecific interactions remain constant across different treatments. This experimental design is suitable for examining the outcome of interspecific competition on the overall impacts on host suppression. If interspecific interactions between parasitoid species have no effect on the host population (i.e., two different parasitoids act independently), host mortality should follow a multiplicative risk model [24,25]

(*P*_exp_ = [*P*_1_ + *P*_2_] − [*P*_1_ × *P*_2_])

where _exp_ is the expected host mortality from both parasitoids together, *P*_1_ is the observed host mortality by parasitoid species 1 alone, while *P*_2_ is the observed host mortality by parasitoid species 2 alone. If the observed (O) and expected (E) parasitism is no different, there is no negative impact), if O > E, the impact on host mortality is additive, and if O < E, the impact on host mortality is negative.

### 2.7. Statistical Analysis

An analysis of variance was used to assess the potential significance of differences in the number of parasitoids produced by parasitism on the different hosts exposed to the wasps. Frequency of visits and parasitism were statistically analyzed using one-way ANOVA. Prior to the ANOVA, data were checked for normality and homoscedasticity, and percentage data were arcsine square-root transformed before analysis as needed to normalize the variance. Means were separated by Tukey’s standardized range honestly significant difference test and t-tests at the α = 0.05 level. The observed and expected levels of host mortality were compared across replicates using *t*-tests. All analyses were conducted using SAS JMP Version 11 [26].

## 3. Results

### 3.1. Explorations and Acquiring Parasitoids

**Figure 5:**
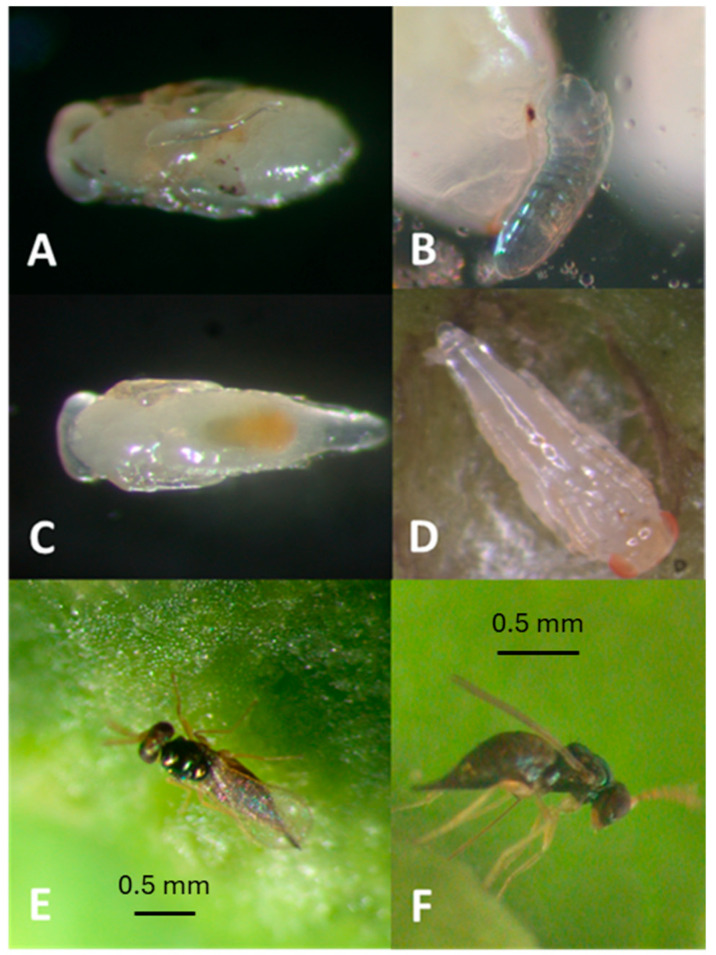
Developmental stages of *Aprostocetus nitens*: (**A**) an egg deposited on pupa of EGW; (**B**) ectoparasitic larva and oviposition scar; (**C**) tapered pupa, dorsal view of female; (**D**) ventral view of pupa; (**E**) female *Aprostocetus nitens* on a EGW gall; (**F**) ovipositing female with long exerted ovipositor.

**Figure 6:**
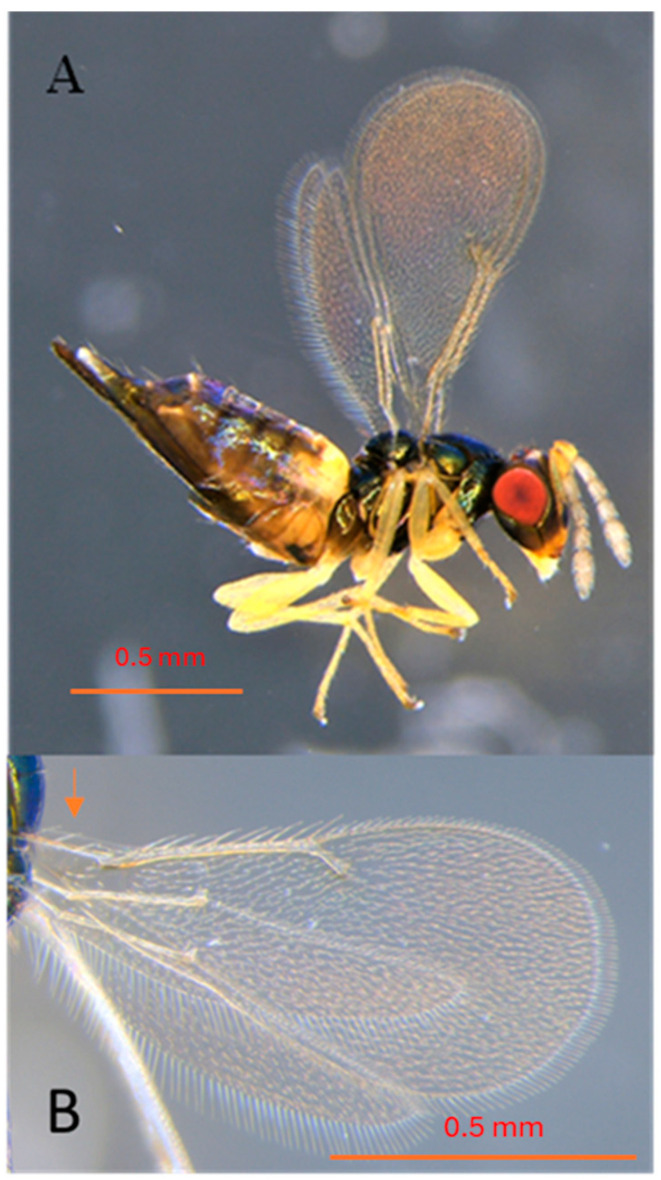
*Aprostocetus nitens* of thelytokous female: (**A**) side view habitus with pointed ovipositor; legs yellow with middle and hind coxae metallic black. Dark tarsal tips; scutellum strongly convex with distinct submedian and sublateral lines; female antenna with three segmented funicles; segment F-III a little shorter than F-I; (**B**) hyaline wings venation pale brown, showing three dorsal setae on the premarginal vein of the front wing (red arrow). Photo credited to Janis Matsunaga (HDOA, Honolulu, HI, USA).

*Erythrina* trees in Africa were healthy with sporadic galls on leaves during the surveys. *Aprostocetus nitens* was first described by Prinsloo and Kelly (2009) [19] as a parasitoid soon after it was discovered in Tanzania and South Africa as a potential biocontrol agent of *Quarastichus* species. *Aprostocetus nitens* is shiny black in color with a dark metallic green tinge and yellow gaster, antennae, and legs (see Prinsloo and Kelly 2009 for full description [19]), Figure 5 and Figure 6.

*Aprostocetus exertus* is known from Tanzania as a primary parasitoid of *Q. erythrinae* and *Q. ingens* in galls of *E. latissima* in South Africa [18,19]. *Aprostocetus nitens* and *A. tritus* are parasitoids of the *Quadrastichus* gall wasps and have been reared in association with *Q. bardus*, *Q. erythrinae*, and *Q. gallicola*. *Aprostocetus nitens* head and body black with a dark metallic green tinge (Figure 6). *Aprostocetus tritus* head and body without a metallic luster, blackish brown except base of gaster yellowish, and submarginal vein of the forewing with four dorsal setae (not reared).

### 3.2. Tri-Trophical Associations of Native Erythrina, Gall Wasps, and Their Parasitoids

**Figure 7:**
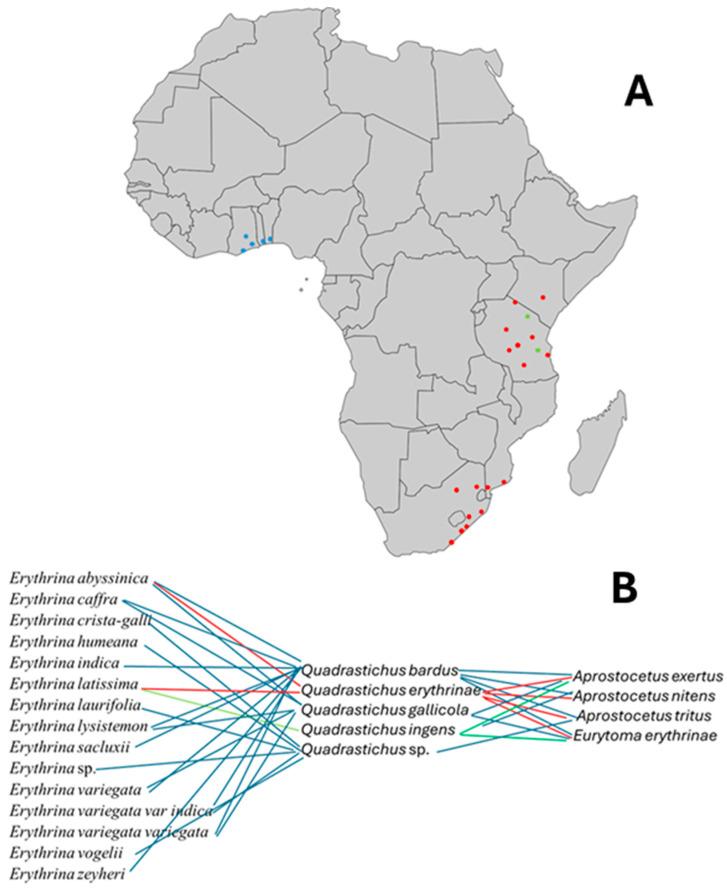
(**A**) Major collection localities of gall wasp parasitoids in West Africa (Benin, Ghana, Togo, with blue points indicating *Aprostocetus exretus*) and eastern Africa (Kenya, Mozambique, South Africa, and Tanzania) (see Table S1 for more location information). (**B**) Tri-trophic associations among 15 *Erythrina* species, 5 gall formers, and 4 parasitoids in east and west Africa (red, green, and blue lines indicate associations for *Q. erythrinae*, *Q. ingens,* and other *Quadrastichus*, respectively).

**Figure 8:**
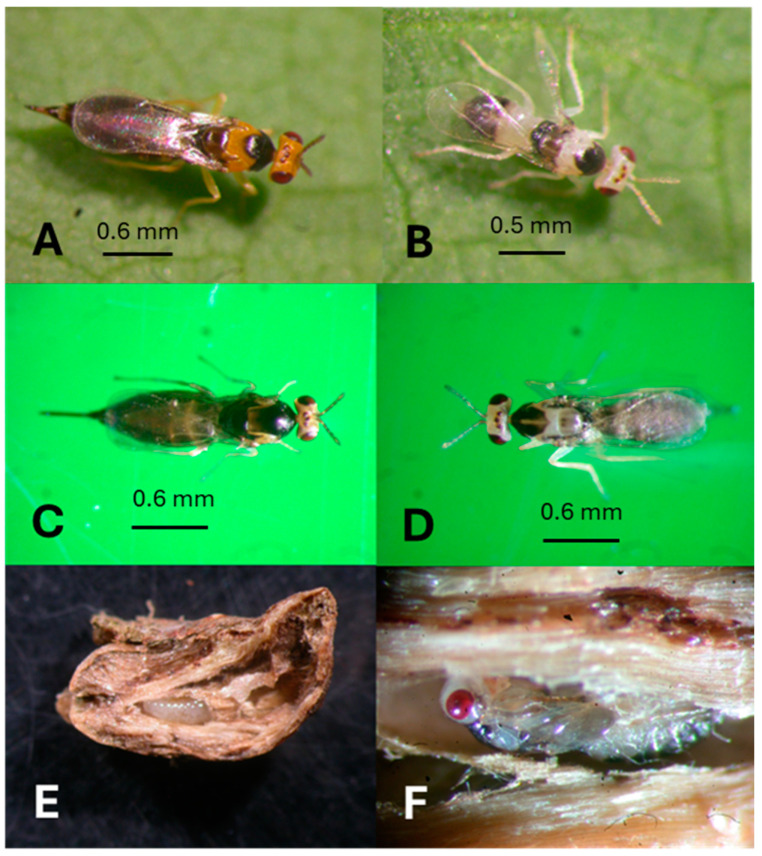
Gall formers in Africa; (**A**) *Quadrastichus erythrinae* female, Tanzania only; (**B**) male *Q. erythrinae*, Tanzania only; (**C**) *Q. ingens* female ex *Erythrina latissima*, South Africa; (**D**) male *Q. ingens*; (**E**) a gall removed from *E. latissima* leaf with *Q. ingens* larva in a woody chamber cavity (horizontal figure, 0.4 Ø × 1.5 cm height); (**F**) pupa of *Q. ingens* in a woody chamber cavity, South Africa.

Fifteen species of native African *Erythrina* were examined for galls (Table S1). Five species of gall inducers and four endemic natural enemies were recognized (Table S1, Figure 7A). *Erythrina abyssinica* from Tanzania and *E. lysistemon* from South Africa were mostly galled by *Quadrastichus bardus*, and *Q. gallicola*. *Quadrastichus ingens* was found only in association with *Erythrina latissima* from South Africa (Figure 8C–F). Initial infestations by *Quadrastichus erythrinae* were detected in Arusha, Bwawani, Iringa, and Morogoro (Tanzania). These were confirmed by La Salle (CSIRO, Pullenvale, QLD, Australia) based on morphological characters (Figure 8A,B). The third natural enemy, *A. exertus*, was reared for four generations under Hawaii ICF conditions and died owing to extensive male progeny produced in the colony (Figure 4C).

A tri-trophic association with the 15 surveyed *Erythrina* species, and five different gall formers were linked to four parasitoids in Figure 7B. *Aprostocetus nitens* was the dominant parasitoid emerging from *Q. bardus*, *Q. erythrinae*, and *Q. gallicola* from eastern Africa only. Followed by the *Eurytoma erythrinae* as the second major parasitoid from eastern and western Africa (Figure 4D). *Aprostocetus exertus* was present in Benin, South Africa, and Tanzania (Figure 4C). In South Africa, *Q. ingens* is being parasitized by *A. exertus* and *E. erythrinae*. From the food chain figure, *Q. erythrinae* was emerging from samples of *E. abyssinica* and *E. latissima* collected only from the Tanzanian leaf samples from Bwawani, Chalinze, Gweta, and Rubungo villages (Figure 8A,B). Those samples were heavily parasitized by the three common parasitoids and mostly by *A. nitens. Aprostocetus exertus* emerged from Benin samples along with the regular parasitoid *Eurytoma erythrinae* in association with *Q. bardus* and *Qudrastichus* sp. from galls of *E. indica*, and *E. vogelii* (Table S1).

### 3.3. Longevity and Life-Time Fecundity of A. nitens

Biological studies of *A. nitens* were performed at the HDOA-ICF. This species reproduces parthenogenetically (via thelytoky parthenogenesis, the absence of mating and subsequent production of all female diploid offspring) under laboratory conditions. However, the first generation of field collections of this species from South Africa and Tanzania produced both sexes.

**Figure 9:**
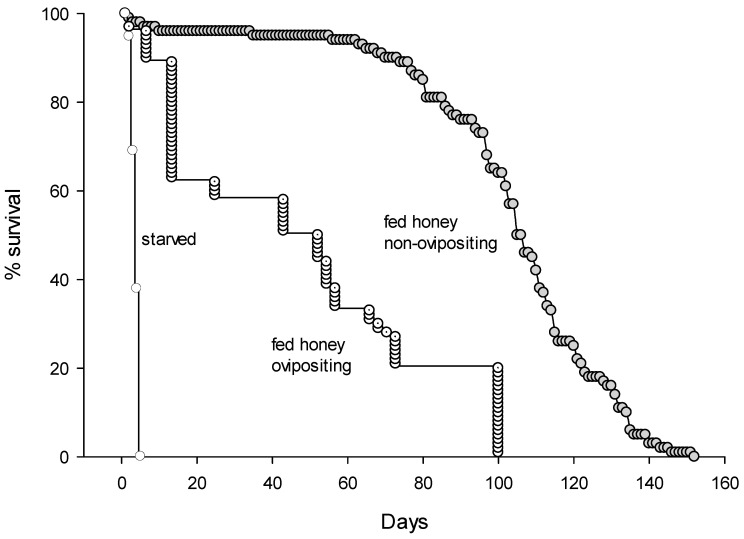
Survivorship curves of starved, ovipositing, and non-ovipositing (fed honey) females of *Aprostocetus nitens*.

Eggs are laid on the host larvae or pupae of EGW after stinging (Figure 5A). It took an average of three days for eggs to hatch. Larvae matured after 11 days (Figure 5B) confined in galls feeding on EGW larvae. The pupal stage took up to 6 days (Figure 5C,D). Adult females start to lay eggs after three preoviposition days (Figure 5E,F). The entire life cycle for this species, from egg to adult, took 20.1 ± 0.28 days, and newly hatched female offspring contain one or two mature eggs in their ovaries. Females are synovigenic, producing an average of 156.7 ± 22.3 offspring throughout their lifespan. This species can survive 4.0 ± 3.0 days without food, and non-ovipositing females lived significantly longer than ovipositing females for 102.5 ± 2.3 days when fed honey (F_2,199_ = 349.04, *p* < 0.0001). Ovipositing females lived for 46.9 ± 3.5 days (Figure 9) and continued to lay eggs for 25.1 ± 2.3 days, with a rate of 5.9 ± 0.45 offspring per day (Table 1).

### 3.4. Host Specificity of A. nitens

**Figure 10:**
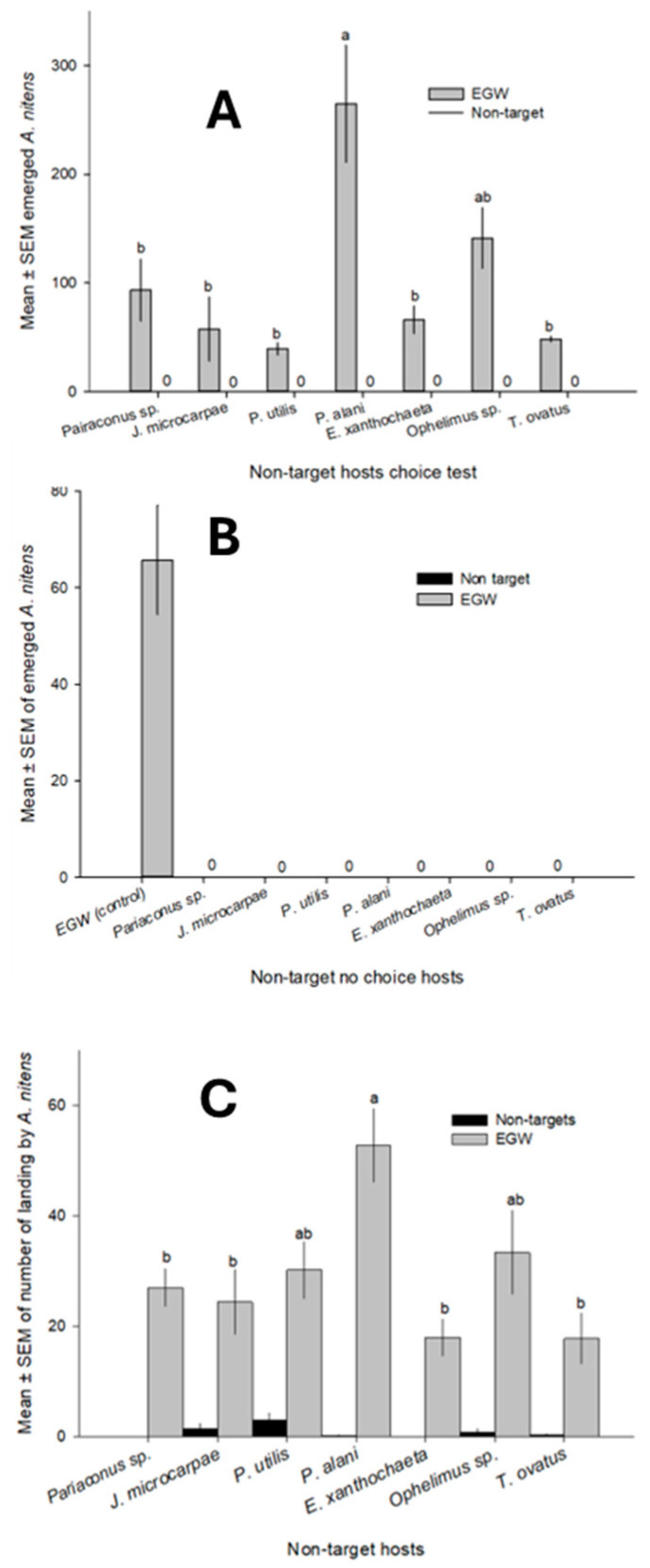
(**A**) Choice tests with *Aprostocetus nitens* and non-targets; (**B**) *A. nitens* emergence from control and non-target hosts in no-choice tests; (**C**) frequency of visits of non-targets by *A. nitens*. Values are mean ± SEM, and bars topped by different letters are significantly different (*p* < 0.05).

In both choice and no-choice host specificity tests, not a single *A. nitens* emerged from any of the seven tested non-target gall-forming species (Figure 10A,B). In contrast, 101.6 ± 15.9 ($\bar{x}$ ± SEM) and 29.08 ± 2.6 wasps emerged from EGW in the choice and no-choice tests, respectively. These results show statistically significant differences between the parasitism of the target host and non-target candidates in all cases (choice test: F_6,28_ = 8.1, *p* < 0.0001; no choice test: F_6,28_ = 8.1, *p* < 0.0001). Although the parasitoid visited or examined some non-target host galls, the frequency of landing on the non-target host galls (0.83 ± 0.28) was significantly lower than on the target host galls (29.08 ± 2.66) (t_68_ = 10.6, *p* < 0.0001). There was no attempt made by the wasps to parasitize the non-target galls. These results suggest that none of these non-target species was recognized as a suitable host substrate by *A. nitens* (Figure 10C).

### 3.5. Interspecific Competition Between A. nitens and E. erythrinae

**Figure 11:**
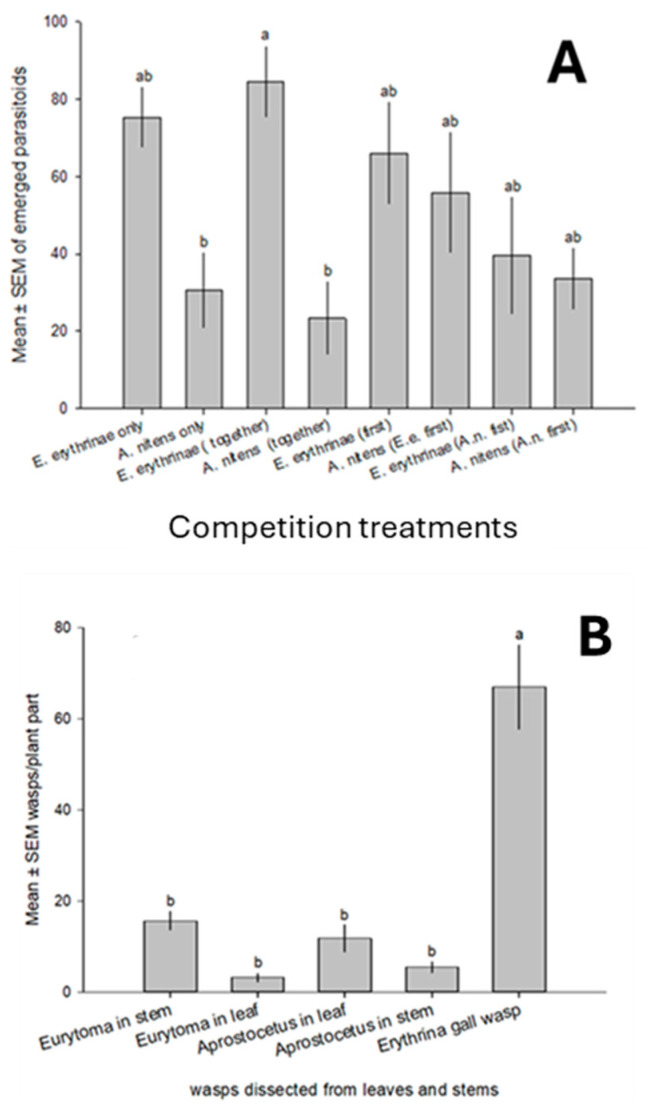
(**A**) Means of emerged parasitoids in competition study between *E. erythrinae* and *A. nitens*; (**B**) mean number of wasps dissected from leaves and stems during the competition tests. Values are mean ± SEM and different letters on top of bars are significantly different (*p* < 0.05).

Overall, more *E. erythrinae* emerged than *A. nitens* in both single and two parasitoid species exposures (Figure 11A). There was no significant reduction in the number of emerged wasps for both parasitoids when they were exposed alone or together to the hosts. Consequently, when the two parasitoids were released concurrently, the total levels of parasitism (84.4 ± 9.2%) would be higher than *E. erythrinae* alone (75.4 ± 7.4%) or *Aprostocetus* alone (33.6 ± 8.8) (F_7,32_ = 3.89, *p* = 0.004). The observed and expected host mortality by both parasitoid species was not different (t_8_ = 1.35, *p* = 0.233), i.e., no negative impact of the interspecific interaction on host suppression. Although there was much lower parasitism by *A. nitens* than *E. erythrinae*, the number of both parasitoid species dissected from leaves and stems during the competition tests was similar, suggesting that a slight preference by *A. nitens* for galls on stems and petioles and by *E. erythrinae* for galls on foliage (Figure 11B).

Field observations in Mozambique, South Africa, and Tanzania indicated that *A. nitens* emerged only from galls produced on several *Erythrina* species, rather than any other host plants considered during field work. Such galls may contain the *Erythrina* gall wasp, *Q. erythrinae*, or other African *Quadrastichus* species. This putative limited host range was confirmed by these experiments performed at the HDOA ICF.

## 4. Discussion

*Aprostocetus* is one of the largest of all chalcid genera of the subfamily Tetrastichinae (Hymenoptera: Eulophidae) with about 813 described species globally. They act as parasitoids and gall formers of many species. Many of them are important biocontrol agents of insect pests [27,28,29]. In this study, for the first time, we document *A. nitens* as an important parasitoid for biological control of EGW. We described its ecological interactions with host insects and host trees in its native range and evaluated its host specificity and potential interaction and outcomes with the established *E. erythrinae* for biological control of EGW. Our quarantine screening showed host specificity on EGW. Seven gall inducer non-target species were tested with apparently no attempted or successful attack or development. The target host was included in all replicates of non-target testing to demonstrate *A. nitens* competence, i.e., to show that wasps used in each test were healthy and the environmental conditions were suitable for successful parasitism. Thus, both field observations in its native range and quarantine evaluations in Hawaii showed that *A. nitens* is highly specific to EGW, and strongly indicate that the proposed release of this parasitoid for the biocontrol of EGW will unlikely cause undesirable, negative, non-target effects in the natural environment of the Hawaiian Islands.

Understanding and predicting potential competitive outcomes is important in the design of classical, augmentative, and conservation biological control programs when considering multiple species introductions [30]. Based upon the experimental data obtained in this study, the proposed release and establishment of *A. nitens* is expected to supplement the positive impacts of *E. erythrinae* in suppressing infestations of EGW. Interspecific competition between these two parasitoids may still occur if both attack limited hosts in old leaves and stems, but they seem to have slightly different preferences for host feeding niches, which can facilitate their co-existence and improve overall impacts on EGW. Releasing *A. nitens* would likely increasingly affect immature EGW stages in these young leaves, flowers, and seed pods. This should result in increased seed set and seed maturation and positively improve the opportunity for recruitment of the endemic *Erythrina*.

The higher fecundity rate of *A. nitens* compared to *E. erythrinae* may also confer an advantage upon the former species over the latter. Indeed, the dominance of *A. nitens* over the established parasitoid in the field, as seen from the African surveys, may be related to its high fecundity and likely plasticity of body growth and development on differently sized hosts. This phenomenon may be observed in several parasitoids in the field in Hawaiʻi. *Psyttalia humilis* (Silvestri) (Hymenoptera: Braconidae), a South African parasitoid of fruit flies (Diptera: Tephritidae), became rare after being released in Hawaii with the Australian parasitoid, *Diachasmomorpha tryoni* (Cameron), for biocontrol of *Ceratitis capitata* (Wiedemann). While *D. tryoni* contributed to the reduction of *C. capitata* populations, *P. humilis* died out [31,32,33]. This was attributed to the superior fecundity of *D. tryoni* [34,35]. The invasion of *Bactrocera dorsalis* (Hendel) in the 1950s led to the introduction of several opiine parasitoid species from Asia to Hawaiʻi [31,36,37]. Among them, *Diachasmimorpha longicaudata* (Ashmead) became widely established, but thereafter was largely replaced by *Fopius vandenboschi* (Fullaway) [38,39]. Subsequently, the abundance of these two species declined following the introduction and successful establishment of *Fopius arisanus* (Sonan) [38]. *Fopius arisanus* reproductive superiority makes it the dominant opiine on the islands, resulting in 74% of the total parasitism of *C. capitata* [32]. The abundance of *D. tryoni* also sharply declined (to <4.0% parasitism) after *F. arisanus* became established in *C. capitata* populations. Observations during the 1950s suggested that dominance and displacement of parasitoids were influenced by the superior competitive ability of species with high intrinsic rates of population increase [32,38,40] as well as competitive superiority by the early acting egg parasitoid [41]. We expect *Aprostocetus* to coexist with *E. erythrinae*, likely filling slightly separate feeding niches.

We do not know the causes of the thylotokous development of this parasitoid in our quarantine rearing, which warrants further study. However, environmental conditions may stimulate the wasps to revert to sexual reproduction after the release. For example, thelytokous reproduction in *Trichogramma* spp. (Hymenoptera: Trichogrammatidae) can change to arrhenotoky under exposure to antibiotics or high temperatures (>30 °C) under experimental rearing conditions. This results in a significant increase in the percentage of male offspring and a decrease in the percentage of female offspring in wild populations [30].

Other than Hawaiʻi, only Taiwan and Japan have implemented biocontrol of EGW. Japan imported *E. erythrinae* from Hawaiʻi, but no recent reports on the outcome of the releases have been published. In Taiwan, resident parasitoids were found to attack EGW, one of which was *Aprostocetus felix* LaSalle, Yang & Lin, a new species to science [14]. *Aprostocetus felix*, a solitary parasitoid collected from mature larvae and pupae, dominated the other local parasitoids of *Q. erythrinae*. Population levels of this species remain high in cooler times of the year when other parasitoid population levels have declined. The parasitoid populations have built up through the years in Taiwan and may serve as a biological control agent of EGW there [3].

## 5. Conclusions

To examine the community-wide effects of *A. nitens* in Africa, we constructed a food web chart showing interactions among 15 *Erythrina* species, five *Quadrastichus* spp. gall formers, and their four major parasitoids, three *Aprostocetus* spp. (Eulophidae) and *Eurytoma erythrinae* (Eurytomidae).

Field observations in Tanzania and host specificity tests in HDOA ICF provide strong support for the hypothesis that *A. nitens* will not attack non-target gall-forming species, and the parasitoid should be approved for field release in Hawaiʻi. Competition studies showed *A. nitens* will be unlikely to negatively affect *E. erythrinae* but is likely to contribute to the overall suppression of EGW owing to their different preference for host feeding niches. The high fecundity and longevity of *A. nitens* are also important attributes for a biological control agent for EGW. We predict that *A. nitens* will be a valuable addition to EGW control programs in Hawaiʻi and potentially other places.

## Figures and Tables

**Table 1:** Reproductive attributes of thelytokous *Aprostocetus nitens* females.

Reproductive Parameter	n	Mean ± SEM	Range
Survival of non-ovipositing females, fed honey (days)	100	102.5 ± 2.3 a	1–152
Survival of ovipositing females, fed honey (days)	44	46.9 ± 3.5 b	22–97
Survival of starved females (days)	58	4.0 ± 3.0 c	2–5
Oviposition period (days)	7	25.1 ± 2.3	17–34
Realized fecundity	7	156.7 ± 22.3	107–282
Daily progeny	216	5.9 ± 0.45	0–53
Female lifecycle (days)	11	20.1 ± 0.28	19–22

Ovipositing females were provided EGW-infested plants once every 7 days until females died. Means followed by different letters are significantly different (F_2,199_ = 349.04, *p* < 0.0001, Tukey’s HSD test).

## Data Availability

The original contributions presented in this study are included in the article/Supplementary Material. Further inquiries can be directed to the corresponding author.

## Supplementary Materials

The following supporting information can be downloaded at: https://www.mdpi.com/article/10.3390/insects16050519/s1, Table S1: Diversity and distribution of the different species of Hymenopterous (Eulophidae) gall formers and their parasitoid assembly recovered from *Erythrina* species in east and west Africa during surveys in 2005–2007; Figure S1: *Aprostocetus nitens* female: (A) mesothorax showing spherical scutelum with a pair of setae on each side. Setae on mesothorax adfrontal setae and suture; (B) side view of female showing three dorsal setae on premarginal vein (red arrow), setose head and clear eye; (C) female antenna showing annelli and three yellow funicular segments longer than wide; Figure S2. Male *Aprostocetus nitens*: (A) dorsal habitus showing metallic mesothorax, pale basal half of gaster, and pointed male genetalia; (B) side view showing yellow legs, pale basal half of gaster; (C) hairy four yellow funicular segments, 3 segmented clava, and pedicel; (D) ventral shiny brown plaque on ventral margin of scape, visible as a distinct dark subapical patch on ventral margin of scape (red arrow).
