# Supplementary material for: Aprostocetus nitens (Hymenoptera: Eulophidae), an Ectoparasitoid Proposed for Biological Control of the Destructive Erythrina Gall Wasp, Quadrastichus erythrinae, in Hawaiʻi
Source: Insects. 2025 May 14;16(5):519. doi: 10.3390/insects16050519 (PMC12112616; doi:10.3390/insects16050519)
Supplement: Supplementary file 1 [file insects-16-00519-s001.zip › insects-3331642-supplementary.pdf]

Supplementary file

Table S. 1. Diversity and distribution of the different species of Hymenopterous (Eulophidae) gall formers and their parasitoid assembly recovered from *Erythrina* species in east and west Africa during surveys in 2005 – 2007.

**Country**	**Collection locality (GPS, elevation)**	**Dates of survey**	***Erythrina* species (mean ± SEM number of galled leaves collected)**	**Gall wasp species**	**Associated parasitoids**
Benin	Porto-Novo (6°28′46.4″ N, 2°37′13.13″ E, 16 m)	28 May –2 Jun. 2006	[5]*Erythrina indica* (36.1 ± 6.1, *n* = 18)<br>[10]*Erythrina* sp. (102.8 ± 25.0 (*n* = 7)<br>[14]*Erythrina vogelii* (54)	[a]*Quadrastichus bardus*<br>[e]*Quadrastichus* sp.	[a]*Aprostocetus exertus*<br>[a]*Eurytoma erythrinae*
Ghana	Adomi-mamiwata Eastern region,<br>Juapong (6°17′36.42″ N, 0°12′03.38″ E, 58 m), Akuamanji to Akogombo, Nyanta, Chechlim, Elmina (5° 06′18.66″ N, 1°20′31.74″ W, 17 m), Atitetu	4–7 Jun. 2006	[10]*Erythrina* sp. (101.7 ± 33.1, *n* = 6)	[e]*Quadrastichus* sp.	[e]*Eurytoma erythrinae*
Kenya	Runyejes, Embu (0°29′06″ S, 37°35′13″ E, 1281 m)		[10]*Erythrina* sp. (384)	[a]*Quadrastichus bardus*<br>[e]*Quadrastichus* sp.	[a]*Aprostocetus nitens*<br>[a]*Eurytoma erythrinae*
Mozambique	Nmaacha Maputo (25°59′08.87″ S, 32°02′27.43″ E, 489 m), Tavira village, Jarden Tendora, Parue Jose Carbran, Parue Jose Carbran, Bilene	17–30 Mar. 2007	[4]*Erythrina humeana* (114)<br>[7]*Erythrina laurifolia* (161)<br>[8]*Erythrina lysistemon*<br>[10]*Erythrina* sp. (342)	[a]*Quadrastichus bardus*	[a]*Aprostocetus nitens*<br>[a]*Eurytoma erythrinae*
South Africa	Nelspruit (25°28′33.66″ S, 30°58′07.67″ E, 673 m), Mpumalanga	12 Dec. 2005	[10]*Erythrina* sp. (65)	[c]*Quadrastichus gallicola*	[c]*Aprostocetus nitens*<br>[c]*Eurytoma erythrinae*
South Africa	Elysium, southcoast Durban, Hibberdene Durban, Durban Botanical Garden (29°50′53.66″ S, 31°00′29.77″ E, 15 m), Camperdown, Skukuza, Nelspruit, Lowvelt Botanical Garden, Nelspruit Mataffin Hill, Tamboti nr Shayamoya,	16 Jan. – 26 Jun. 2006	[2]*Erythrina caffra* (92.8 ± 39.3, *n* = 6)<br>[3]*Erythrina crista-galli* (22.0 ± 5.4, *n* = 4)<br>[4]*Erythrina humeana* (36)<br>[6]*Erythrina latissima* (49.5 ± 34.5, *n* = 2)<br>[8]*Erythrina lysistemon* (96.3 ± 17.7, *n* = 24)<br>[10]*Erythrina* sp. (17.6 ± 8.8, *n* = 4)	[a]*Quadrastichus bardus*<br>[c]*Quadrastichus gallicola*<br>[d]*Quadrastichus ingens*	[ac]*Aprostocetus nitens*<br>[c]*Aprostocetus tritus*<br>[a]*Eurytoma erythrinae*

	memorial gate, Huhluwe, Empangeni, Durban harbor, Pietermaritzberg Botanical Garden, East London, Grahamstown center, Vergelegen Estate, Somerset West, Durban Tollgate, Road to Cato Manor, Francois Rd Durban, Tinely Manor (29°25′36.99″ S, 31°16′20.72″ E, 65 m), Camperdown, Durban Botanical Garden				
South Africa	Camperdown (29°43′ 45.36″ S, 30°31′54.52″ E, 758 m MAMSL), Scottburgh, Pietermaritzburg, Pretoria Botanical Garden (25°44′22.30″ S, 28°16′23.91″ E, 1356 m), Hazeyview,	12 Feb. – 9 Apr. 2007	[2]*Erythrina caffra*<br>[4]*Erythrina humeana*<br>[6]*Erythrina latissima* (42.5 ± 17.5, *n* = 2)<br>[8]*Erythrina lysistemon* (160)<br>[15]*Erythrina zeyheri*	[c]*Quadrastichus gallicola*<br>[d]*Quadrastichus ingens*	[d]*Aprostocetus exertus*<br>[c]*Aprostocetus nitens*<br>[c]*Aprostocetus tritus*<br>[d]*Eurytoma erythrinae*
Tanzania	Chalinze, Morogoro (6°38′20.07″ S, 38°21′ 10.74″ E, 211 m)	31 Dec. 2005	[1]*Erythrina abyssinica* (40 ♀, 9 ♂)	[b]*Quadrastichus erythrinae*	[b]*Eurytoma erythrinae*
Tanzania	Kilolo village Iringa (8° 00′21.88″S, 35°50′36.42″ E, 1860 m MAMSL), Kibaone village Iringa, Itamba village, Iringa (7°45′05.10″ S, 35°40′ 15.49″ E, 1517 m MAMSL), Tarime, Masumo , Mwanza, Elemera village (2° 31′ 00.15″ S, 32° 55′ 02.79″ E, 1258 m), Kiumba, Kisase (2°33′03.74″ S, 33°01′59.04″ E, 1271 m), Iringa, Kibiki, Manyara Kilimantembo, Arusha, Maroron village, Masai camp village (3°23′06.66″ S, 36°43′13.43″ E, 1340 m), Nganza, Ihayabuyaga , Ilendeje, Mwanza	5 Jan. – 19 Feb. 2006	[1]*Erythrina abyssinica* (311.8 ± 77.2, *n* = 7)<br>[6]*Erythrina latissima* (142.0 ± 50.6, *n* = 6)<br>[9]*Erythrina sacluxii* (19)<br>[10]*Erythrina* sp. (191.3 ± 97.3, *n* = 3)<br>[11]*Erythrina variegata* (55)<br>[13]*Erythrina variegata variegata* (58.3 ± 12.7, *n* = 3)	[a]*Quadrastichus bardus*<br>[b]*Quadrastichus erythrinae*<br>[c]*Quadrastichus gallicola*<br>[e]*Quadrastichus* sp.	[ab]*Aprostocetus nitens*<br>[ab]*Aprostocetus exertus*<br>[e]*Aprostocetus tritus*<br>[ab]*Eurytoma erythrinae*

Tanzania	Bwawani (3°34′53.61″ S, 36°46′41.37″ E, 977 m), Maseyu, Rubungo (1°55′30.68″ S, 30° 09′02.39″ E, 1657 m MAMSL), Dar essalam, Iringa, Masai Camp Arusha, Elemela, Mwanza, Kisase, Nganza, Luchelele , Gweta Morogoro (20° 11′26.50″ S, 25°15′ 18.91″ E, 929 m)	31 Jan. – 19 Feb. 2007	[1]*Erythrina abyssinica* (142.3 ± 18.1, *n* = 17)<br>[11]*Erythrina variegata*<br>[12]*Erythrina variegata var indica* (193)	[a]*Quadrastichus bardus*<br>[b]*Quadrastichus erythinae*	[ab]*Aprostocetus nitens*<br>[ab]*Aprorstocetus exertus*<br>[a]*Aprostocetus tritus*<br>[ab]*Eurytoma erythrinae*
Togo	Atchanre, Devie tsevie, Agbato house, Tsevie, Ahekepe, Tabigbo, Aneho (6°13′39.09″ N, 1°34′54.28″ E, 3.9 m)	11–12 Jun. 2006	[10]*Erythrina* sp. (208.8 ± 110.9, *n* = 6)	[e]*Quadrastichus* sp.	[e]*Eurytoma erythrinae*

Collections made by M. Ramadan, HDOA (Mozambique, South Africa, Tanzania), Affi ICIPE (Kenya), Dan Rubinoff, Mark Wright, and Russell Messing, UH (South Africa), T.G. Gruit and J. Daneel, SA Citrus Research International, and Aimé Bokonon-Ganta, UH (Benin, Ghana, Togo). Superscript numbers are the *Erythrina* species, while superscript letters are the different *Quadrastichus* species and associated parasitoids in samples.

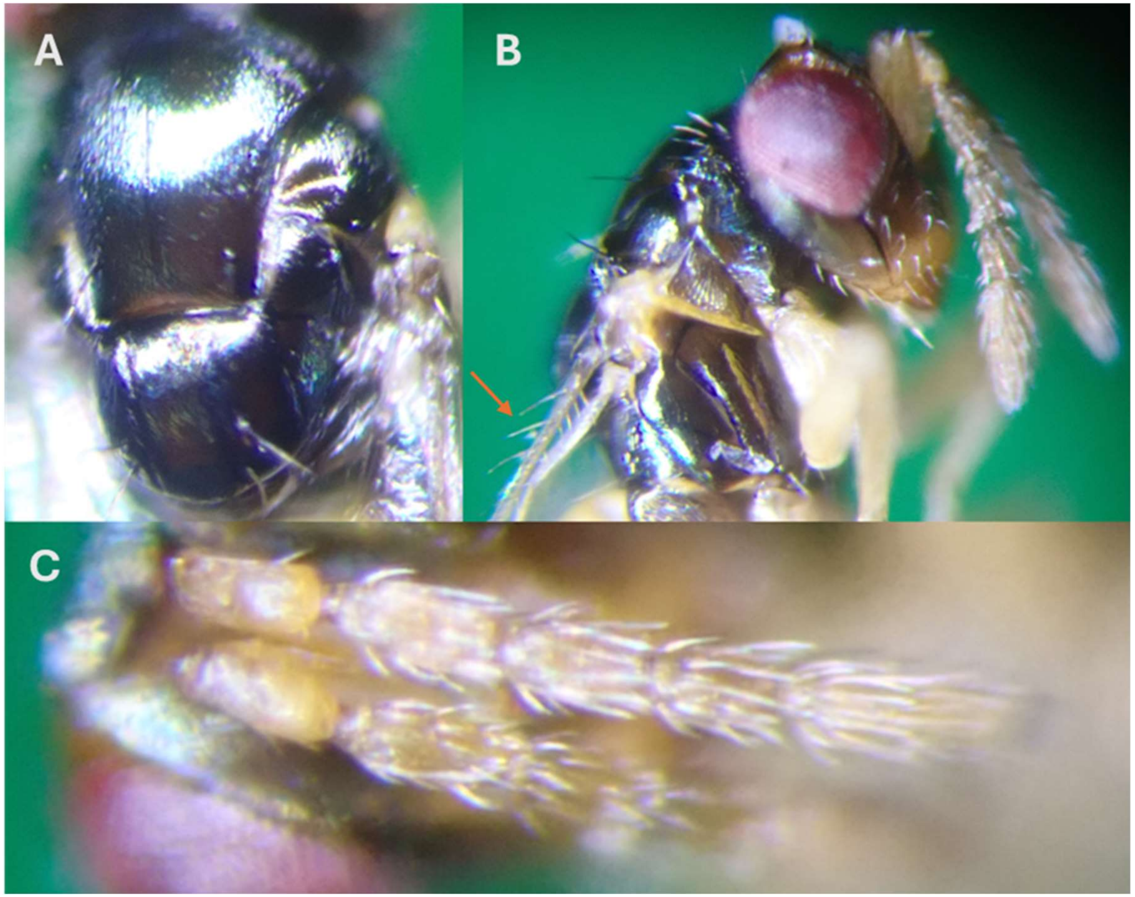

Figure S 1. *Aprostocetus nitens* female: A) mesothorax showing spherical scutelum with a pair of setae on each side. Setae on mesothorax adfrontal setae and suture; B) side view of female showing three dorsal setae on premarginal vein (red arrow), setose head and clear eye; C) female antenna showing annelli and three yellow funicular segments longer than wide.

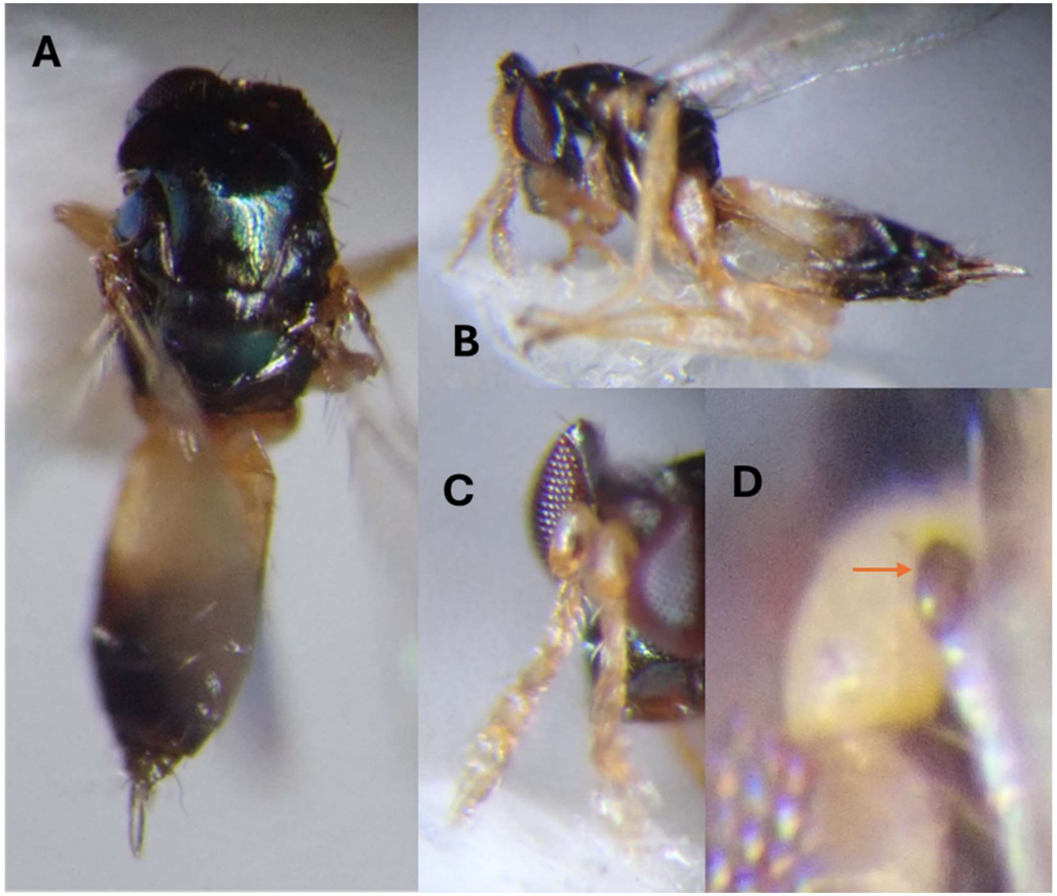

Figure S 2. Male *Aprostocetus nitens*: A) dorsal habitus showing metallic mesothorax, pale basal half of gaster, and pointed male genetalia; B) side view showing yellow legs, pale basal half of gaster; C) hairy four yellow funicular segments, 3 segmented clava, and pedicel; D) ventral shiny brown plaque on ventral margin of scape, visible as a distinct dark subapical patch on ventral margin of scape (red arrow).
